# Fellow-Workers and the Roll of Honour

**Published:** 1914-12-19

**Authors:** 


### ROUND THE WORLD.

[The Editor Invites Early Information and Contributions Marked "Fellow-Workers."]

### Fellow-Workers and the Roll of Honour.

The twelve physicians and surgeons who have been appointed as the consulting staff of the new hospital under the Red Cross Society, and whose names follow, constitute a galaxy of eminent professional men such as is seldom associated with any one institution. These distinguished consultants to the King George Hospital at Waterloo enjoy a great reputation for both clinical work and individual research.

Sir Thomas Barlow, the President of the Royal College of Physicians, has won fame not only as a great consulting physician, but as an authority on children's diseases, his clinical researches having been carried out during many years' association with University College Hospital and the famous Great Ormond Street Hospital for Sick Children. A physician-extraordinary to His Majesty the King, he was made a baronet in 1901.

Sir William Watson Cheyne, Bart., M.D., F.R.C.S., F.R.S., the eminent King's College Hospital consultant and President of the Royal College of Surgeons, was mentioned in these notes recently in connection with his appointment as lieutenant-colonel in the Territorial branch of the R.A.M.C.

Sir Rickman John Godlee, Bart., K.C.V.O., F.R.C.S., honorary surgeon in ordinary to the King, and lately President of the Royal College of Surgeons, was for many years a popular teacher at University College Hospital, and his work, which has on more than one occasion been referred to in The Hospital, is so well known as to need little comment.

Sir James Frederic Goodhart, M.D., F.R.C.P., whose work on the active staff of Guy's Hospital brought him into close contact with several generations of medical practitioners, was created a baronet three years ago. He was for many years on the staff of the Evelina Hospital for Sick Children, his observations in connection with children's diseases having been embodied in a well-known text-book on that subject.

Sir Dyce Duckworth, another leading medical consultant and member of the consulting staff of St. Bartholomew's Hospital, has been a prominent figure in the medical profession for a very long time, having been knighted as far back as 1886. When the staff of the Seamen's (*Dreadnought*) Hospital at Greenwich was reconstituted some years ago he was elected one of the physicians. The holder of many academic distinctions and the recipient of innumerable honours, Sir Dyce Duckworth was made a baronet in 1909.

Dr. John Mitchell Bruce, M.D., F.R.C.P., is consulting physician to Charing Cross Hospital, to the Brompton Hospital for Consumption, and to the King Edward VII. Sanatorium at Midhurst. A physician who has for many years enjoyed an extensive consulting practice, he is the author of a very widely read text-book on "Materia Medica and Therapeutics," the circulation of which is understood to have reached over fifty thousand copies. Dr. Mitchell Bruce is senior censor to the Royal College of Physicians and President of the Medical Society of London.

Sir Henry Morris, Bart., F.R.C.S., is a past President of the Royal College of Surgeons and consulting surgeon to Middlesex Hospital. An investigator of great breadth of thought, his ideas of recent years have found literary expression in important fields outside the immediate connections of surgery. He is the author of various well-known publications on surgery and anatomy.

Mr. Edmund Owen, M.B., F.R.C.S., consulting surgeon to St. Mary's Hospital, is a well-known authority on the surgical treatment of various children's diseases and a prominent member of the consulting staff of the Great Ormond Street Hospital. He has also served in the French Hospital for a long time, his work in connection therewith having brought him the decoration of Légion d'honneur. In earlier years a keen exponent of outdoor sport, especially cricket, his interest and support of games, in addition to his interest in their professional welfare, particularly endeared Mr. Owen to the many students who passed through his hands at St. Mary's.

Sir Seymour John Sharkey, M.D., F.R.C.P., who was knighted in the early part of this year, is consulting physician to St. Thomas's Hospital and medical referee to the Treasury. The various important offices he has held include those of censor and senior censor to the Royal College of Physicians, the Presidency of the Neurological Society, and examinerships to various qualifying bodies. Sir Seymour Sharkey has delivered the Goulstonian and Bradshaw lectures, and rendered many important contributions to current pathological and medical literature.

Sir Horatio Bryan Donkin, M.D., F.R.C.P., consulting physician to the Westminster Hospital, has done important work as medical adviser to the Prison Commission and director of Convict Prisons. Formerly he held the appointments of physician to the East London Hospital for Children and lecturer on medicine at the West London School of Medicine for Women. For his public services he was knighted three years ago. Recently Sir Bryan Donkin has occasioned much interest by his challenge to Sir Oliver Lodge to produce scientific evidence of the validity of spiritistic phenomena.

Sir Bertrand Dawson, K.C.V.O., F.R.C.S., physician in ordinary to His Majesty and physician to the London Hospital, has been lately mentioned in these columns in connection with the Territorial R.A.M.C., wherein on mobilisation he held the rank of captain in the 2nd City of London Hospital.

Mr. J. Warrington Howard, F.R.C.S., whose name completes our brief review of the consulting staff of the new King George Hospital, is consulting surgeon to St. George's Hospital, and was at one time President of the Medico-Chirurgical Society, in the days before the foundation of the Royal Society of Medicine. Mr. Warrington Howard holds various appointments and is interested in many philanthropic concerns. He has for some time resided at his place in Hertfordshire.

